# The Effects of SIRT1 on Alzheimer's Disease Models

**DOI:** 10.1155/2012/509529

**Published:** 2012-12-03

**Authors:** Gizem Donmez

**Affiliations:** Department of Neuroscience, Tufts University School of Medicine, Boston, MA 02111, USA

## Abstract

Sirtuins are highly conserved NAD^+^-dependent enzymes that were shown to have beneficial effects against age-related diseases. Aging is the major risk factor for all neurodegenerative disorders including Alzheimer's Disease (AD). Sirtuins have been widely studied in the context of AD using different mouse models. In most of these studies, overexpression of SIRT1 has been shown to have protective effects against AD. Therefore, designing therapeutics based on increasing SIRT1 activity might be important for investigating the ways of treatment for this disease. This paper summarizes the recent research on the effect of SIRT1 in AD animal models and also the potential of SIRT1 being a therapeutical target for AD.

## 1. Introduction

Life expectancy of humans has been doubled in the last century. In 1840s, the life expectancy was around 45 years of age, while in the year 2000, it increased to the age of 80 (World Health Organization reports, 2009). This aged population carries a lot of age-related diseases including neurodegeneration. Alzheimer's Disease (AD) is the most common neurodegenerative disorder resulting in memory loss, cognitive decline, functional decline, and death. Pathognomonic signs of AD are extracellular amyloid plaques and intracellular neurofibrillary tangles. Although many advances in identifying the molecular mechanisms involved in AD have been made, there is still no cure or treatment available for this disease. 

Genetic and nongenetic factors are among the important causes of AD, with aging playing a major role. Therefore, any molecular mechanism that delays or interferes with the age-related decline in brain might delay or prevent AD. Sirtuins are a highly conserved class of protein deacetylases, which are age-related molecules [1] and may have beneficial effects against age-related diseases [1]. They are lysine deacetylases that remove acetyl groups from lysines by hydrolysis, both on histones and nonhistone proteins. Although there are seven human orthologs (SIRT1 to SIRT7) of yeast proteins, human SIRT1 is the most extensively studied sirtuin in mammalian. Increasing evidence demonstrated the important roles of SIRT1 in AD using different animal models. SIRT1 displays protective effects in mouse models by deacetylating different target proteins and activating different mechanisms. These findings encourage potential efforts for drug discovery and designing therapeutics based on SIRT1 research. 

In this paper, we will focus on the effects of SIRT1 on AD mouse models. However, sirtuins were also shown to play roles in Parkinson's Disease (PD). Genetic analysis of SIRT1 gene promoter suggests that genetic variants within the SIRT1 gene promoter may repress SIRT1 gene expression, contributing to sporadic PD as a risk factor. Overexpression of SIRT1 was shown to reduce alpha-synuclein aggregates in A53T alpha-synuclein model [1]. In addition, SIRT2 inhibitors rescue alpha-synuclein-mediated toxicity in both in vitro and Drosophila model of PD [1]. 

## 2. SIRT1 and Amyloid Plaque Forming Models

Insight into the formation of A*β* plaques has come from studies of the rare familial form of early onset AD [2]. Dominant mutations have been found in the gene encoding the neuronal membrane protein amyloid precursor protein (APP). APP is cleaved in two sequential steps by the *β*- and *γ*-secretases to generate A*β* 1–40 and 1–42 amyloid peptides, respectively [3]. A second class of dominant mutations giving rise to familial AD fall in genes presenilin 1 and 2 (PS1 and 2), which encode the components of the *γ*-secretase complex [4] and sequential cleavage of APP by the *β*- and *γ*-secretases leads to accumulating *β*-amyloid (A*β*) peptides and plaques. The production of A*β* peptides is avoided by an alternate APP cleavage pathway mediated by the *α*-secretase followed by the *γ*-secretase [5]. Indeed, *α*-secretase cleavage of APP has been shown to be protective in AD models [6, 7]. 

APPswe/PS1dE9 mice [8] have a nonfunctional PS1 (due to exon 9 deletion) and overexpress the APP Swedish mutation (APPswe). Overexpression of the transgene leads to the development of A*β* plaques at around 3 months of age. Activated microglia and astrocytes are also observed around the plaques. By 6–8 months of age, they develop learning and memory deficits. APPswe/PS1dE9 mice do not display neuronal loss. However, they exhibit clinically relevant AD-like symptoms. These include mild neuritic abnormalities, local plaque-related loss in neuronal activity, increased mortality, high prevalence to unprovoked seizures, and age-dependent deficits in the pre- and postsynaptic cholinergic transmission [9]. Therefore, these mice offer a valuable tool in studies aiming at the development of new therapeutic approaches targeted specifically against the plaques and related neuroinflammation. 

In a recent study, SIRT1 levels were manipulated in APPswe/PS1dE9 mice. SIRT1 was overexpressed or deleted in APPswe/PS1dE9 mice by using SIRT1 overexpressing or SIRT1 brain-specific knockout mouse [10]. In this study, overexpression of SIRT1 reduced A*β* production and A*β* plaques, whereas deleting SIRT1 increased A*β* levels [5]. SIRT1 deacetylates Retinoic Acid Receptor beta (RAR*β*) and activates the transcription of the *α*-secretase ADAM metallopeptidase domain 10 (ADAM10), which increases ADAM10 protein levels in neurons and leads to upregulated APP processing by *α*-secretase [10]. By this way, A*β* production is reduced. SIRT1 overexpression in this mouse model also improved learning and memory deficits, whereas deletion of SIRT1 worsens these phenotypes. Calorie restriction also reduced amyloid plaques in APPswe/PS1dE9 mouse model [11]. The researchers identified the mechanism underlying calorie restriction as the activation of SIRT1. In an in vitro model, SIRT1 protects against microglia-dependent A*β* toxicity through inhibiting Nuclear Factor KappaB (NF-kappaB signaling) [12].

## 3. SIRT1 and Tau Models

Tau is a microtubule-binding protein enriched in neurons, and it promotes the assembly and stabilization of microtubules. Hyperphosphorylation of the tau protein can result in the self-assembly of tangles of paired helical and straight filaments, which are involved in the pathogenesis of AD and other taupathies.

In addition to reducing A*β* plaques, SIRT1 also inhibits the tau-related AD phenotype. Firstly, it was reported that SIRT1 reduction was found to be parallel to tau accumulation [13]. The authors compared the concentration of SIRT1 in the brains of AD patients (*n* = 19) and controls (*n* = 22) using Western immunoblots and in situ hybridization. They reported a significant reduction of SIRT1 mRNA (−29%) and protein (−45%) in the parietal cortex of AD patients, but not in the cerebellum. Further analyses in a second cohort of 36 subjects confirmed that cortical SIRT1 was decreased in AD but not in individuals with mild cognitive impairment.

It has been reported that tau is acetylated at multiple lysine residues and that SIRT1 is able to regulate the level of phosphorylated tau via deacetylation [14]. Furthermore, it was shown that the degradation of phosphorylated tau improves cognitive function and reduces neuronal death in mice; however, when tau is acetylated by the histone acetyltransferase p300, the breakdown of tau is inhibited [14]. In this study, SIRT1 was shown to deacetylate the acetylated tau and consequently reduces its level; conversely, SIRT1 inhibition leads to the opposite effect—increasing the levels of tau and exacerbating the accumulation of pathogenic forms of phosphorylated tau. In supportive of this notion, SIRT1 directly interacts with tau and a deficiency in SIRT1 elevates both acetylated tau and phosphorylated tau in vivo. Moreover, the inhibition of SIRT1 activity blocks tau polyubiquitination and thus the turnover of tau, resulting in the accumulation of phosphorylated tau in neurons. In this study, the tau models used were the transgenic mouse expressing human tau cDNA (1N4R) with P301S mutation [15] and the transgenic mouse expressing the entire human wild-type *MAPT *(hT-PAC-N) with 0N3R and 0N4R as the two predominant tau isoforms [16].

## 4. SIRT1 and Inducible p25 Transgenic Mouse

Mice inducibly overexpressing a toxic coactivator of cyclin-dependent kinase 5 (CDK5), p25, were shown to display a massive degeneration of forebrain with features of AD [17]. Cyclin-dependent kinase 5 (Cdk5) and its regulatory subunit p35 are integral players in the proper development of the mammalian central nervous system. Proteolytic cleavage of p35 generates p25, leading to an aberrant Cdk5 activation. The accumulation of p25 is implicated in several neurodegenerative diseases. In primary neurons, p25 causes apoptosis and tau hyperphosphorylation. Cruz et al. [17] generated inducible transgenic mouse lines overexpressing p25 in the postnatal forebrain. Induction of p25 preferentially directed Cdk5 to pathological substrates. These animals exhibited neuronal loss in the cortex and hippocampus, accompanied by forebrain atrophy, astrogliosis, and caspase-3 activation. Moreover, in these mice, endogenous tau was hyperphosphorylated at many epitopes, thus inducing the aggregation of the accumulated tau protein. Neurofibrillary pathology developed progressively in these animals. In this particular mouse model, resveratrol reduced neurodegeneration in the hippocampus prevented learning impairment and decreased the acetylation of the known SIRT1 substrates PGC-1alpha and p53. Furthermore, the injection of SIRT1 lentivirus in the hippocampus of p25 transgenic mice conferred significant protection against neurodegeneration [18]. 

## 5. Concluding Remarks

Over the last 5 years, our understanding of the role of SIRT1 in AD has expanded vastly. Although, it was recently concluded that there is no real association with SNPs available in that particular study between SIRT1 gene and AD risk in the Finnish population [19], it is still very important to continue searching the potential effect and the related mechanism of SIRT1 in AD. A number of studies have been conducted using different AD mouse models, including A*β* plaque forming models, tau models, and p25 model. There is still a lot to be investigated using these models since genetic manipulation of SIRT1 levels have not been performed using tau or p25 models. Besides this, it would be very interesting to investigate whether other homologs of SIRT1 have any role in AD pathogenesis. Although extensively studied, the biological functions of SIRT1 and other sirtuins remain only partially characterized. Analyzing their roles in a context of disease, such as AD, not only identifies new targets of sirtuins, but also helps us understand their interesting biology and its link to brain pathology. The findings above further support the notion that genes activated by calorie restriction to mediate physiological adaptations, such as sirtuins, may be broadly beneficial in combating diseases of brain aging such as AD. Importantly, SIRT1 activity is enhanced by small-molecule compounds; it may, therefore, be critically important to develop sirtuin activators tailored to cross the blood brain barrier to treat neurodegenerative diseases. The challenge is to be able to harness this protection pharmacologically. Manipulating sirtuin activity pharmacologically may be a fruitful area to improve human health.
